# Diagnostic Performance of the EuroFlow Acute Leukemia Orientation Tube (ALOT) in Pediatric Acute Leukemia: A Single-Center Experience

**DOI:** 10.3390/cancers18132023

**Published:** 2026-06-23

**Authors:** Joanna Bulsa, Łukasz Sędek, Łukasz Słota, Bartosz Perkowski, Tomasz Szczepański

**Affiliations:** 1 Department of Pediatric Hematology and Oncology, Faculty of Medical Sciences in Zabrze, Medical University of Silesia, 40-055 Katowice, Poland; szczep57@poczta.onet.pl; 2 Department of Immunology, Faculty of Medical Sciences in Zabrze, Medical University of Silesia, 40-055 Katowice, Poland; lsedek@sum.edu.pl (Ł.S.); lslota@sum.edu.pl (Ł.S.); bartosz.perkowski@sum.edu.pl (B.P.)

**Keywords:** acute leukemia, flow cytometry, immunophenotyping, pediatric hematology, screening, acute leukemia orientation tube (ALOT), EuroFlow, diagnostic algorithm

## Abstract

Background: Multiparameter flow cytometry is widely used in the diagnosis of acute leukemia, allowing for rapid identification of leukemic cells based on their immunophenotype. The EuroFlow Acute Leukemia Orientation Tube was designed as a standardized screening tool to support early diagnostic orientation and guide further, more targeted testing. In this study, we assessed the diagnostic performance of the ALOT panel in pediatric patients with suspected acute leukemia. Methods: A total of 254 pediatric patients (0–18 years) with suspected acute leukemia were analyzed. Bone marrow samples were assessed using multiparameter flow cytometry with the EuroFlow ALOT panel, comprising eight markers (MPO, cyCD79a, CD34, CD19, CD3, cyCD3, CD7, and CD45). Final diagnoses were established using extended immunophenotypic panels and additional diagnostic methods when required. Samples were processed according to EuroFlow standard operating procedures and acquired on FACSCanto II and FACSCanto 10-color flow cytometers (BD Biosciences). Diagnostic performance was assessed by calculating sensitivity, specificity, precision, accuracy, and negative predictive value. Results: Among 254 patients, 234 were diagnosed with hematologic disorders, while 20 had normal bone marrow findings. The ALOT panel correctly identified all pathological samples and did not misclassify any normal sample, resulting in 100% sensitivity, specificity, positive predictive value, negative predictive value, and accuracy for discrimination between abnormal and normal samples. In terms of exact diagnostic orientation, ALOT correctly classified 244 of 254 cases (96.1%) using a single-tube approach. The remaining 10 cases (3.9%), including rare entities such as Burkitt leukemia, chronic myeloid leukemia, and transient myeloproliferative syndrome, required extended immunophenotypic evaluation. Importantly, these cases were not false negative results, as all were correctly identified as abnormal. Conclusions: The EuroFlow ALOT panel is a reliable screening tool for rapid diagnostic orientation in pediatric acute leukemia. Its implementation facilitates targeted selection of extended immunophenotypic panels, improving the efficiency and cost-effectiveness of diagnostic workflows.

## Simple Summary

Acute leukemia in children requires rapid diagnosis because treatment often needs to start as soon as possible. Flow cytometry is commonly used to identify leukemia cells, but detailed testing can be complex and time-consuming. This study evaluated a simplified screening approach called the Acute Leukemia Orientation Tube (ALOT), which uses a small set of markers to quickly guide the diagnostic process. We analyzed its performance in children with suspected leukemia and assessed its ability to identify the most common disease types. Our findings show that this approach can rapidly distinguish abnormal from normal samples and support the selection of more specific diagnostic tests. This may improve the efficiency of diagnostic workflows, reduce unnecessary testing, and help clinicians reach an early diagnosis more effectively.

## 1. Introduction

Acute leukemias (ALs), including acute lymphoblastic leukemia (ALL) and acute myeloid leukemia (AML), are the most common malignancies in the pediatric population, representing approximately 30% of all pediatric cancers [1,2,3]. Due to their aggressive clinical course, fast and accurate diagnosis is essential to enable the initiation of appropriate treatment [4]. Major improvements in the survival of children with acute lymphoblastic leukemia have been achieved through international collaborative efforts and increasingly precise risk-adapted treatment strategies. These advances rely heavily on accurate and timely diagnostic classification at disease presentation [5]. Multiparameter flow cytometry is a key diagnostic tool in the diagnosis of ALL, allowing for rapid immunophenotypic characterization of leukemic cells using specific surface and intracellular markers [6,7,8,9]. This method demonstrates high sensitivity and specificity, enabling accurate diagnosis and further classification of leukemias [4,10]. Compared to other diagnostic methods, flow cytometry offers wide availability and shorter turnaround time, which is crucial in rapidly progressing hematologic malignancies [11]. Standardized immunophenotypic evaluation has become an essential component of modern leukemia diagnostics, enabling reproducible lineage assignment and disease classification across institutions [7,12]. Molecular methods serve as an important complementary tool in pediatric leukemia diagnostics, allowing for identification of specific genetic mutations and chromosomal abnormalities [13,14]. Although these findings have significant prognostic value and guide therapeutic decisions, molecular analyses are often time-consuming. Therefore, screening approaches such as the Acute Leukemia Orientation Tube (ALOT) play a key role at the initial stage of diagnosis [15,16,17]. The ALOT panel, developed by the EuroFlow Consortium, is a standardized flow cytometric screening tool designed for the initial assessment of bone marrow or peripheral blood involvement in patients with suspected acute leukemia [11,18,19]. It consists of a single-tube antibody combination targeting key markers expressed on leukemic cells of different lineages. The use of ALOT allows for rapid narrowing of the diagnostic possibilities to the most common leukemia subtypes, thereby facilitating the selection of appropriate extended immunophenotypic panels and reducing both diagnostic time and cost. In the context of the EuroFlow Acute Leukemia Orientation Tube (ALOT), the term “diagnostic orientation” refers to the ability of the screening panel to assign a sample to the most appropriate diagnostic category and guide the selection of subsequent disease-specific immunophenotypic panels [18,19]. Diagnostic orientation should therefore be distinguished from definitive diagnosis, which requires comprehensive immunophenotypic, cytogenetic, molecular, and clinical evaluation. Although the diagnostic utility of the EuroFlow ALOT panel has been previously reported [18,19], data regarding its performance in large consecutive pediatric cohorts from routine clinical practice remain limited. Therefore, we evaluated the diagnostic performance of ALOT in a real-world pediatric population referred for suspected acute leukemia, including both common and uncommon hematologic malignancies, as well as patients ultimately found to have normal bone marrow.

## 2. Materials and Methods

### 2.1. Study Population

The study included 254 pediatric patients aged 0–18 years who were hospitalized in the Department of Pediatric Hematology and Oncology in Zabrze (Table 1) with suspected acute leukemia based on clinical symptoms (e.g., lymphadenopathy, weakness, fever) and preliminary laboratory findings (complete blood count and manual peripheral blood smear). All patients underwent bone marrow aspiration biopsy, and samples were analyzed using cytomorphological and flow cytometric methods, as well as additional diagnostic tests when necessary.

### 2.2. ALOT and Flow Cytometry

Immunophenotypic characterization of leukemic cells was performed using multiparameter flow cytometry at the Department of Immunology, Medical University of Silesia in Katowice. Bone marrow aspirates collected in EDTA tubes were processed within 24 h after collection. Samples were stained according to EuroFlow standard operating procedures (SOPs) valid at the time of analysis [11]. For the ALOT panel, intracellular staining was performed using the FIX & PERM Cell Permeabilization Kit (Invitrogen, Carlsbad, CA, USA). For initial diagnostic evaluation, all patients were analyzed using the single-tube ALOT panel. The antibody combination included the following markers: MPO-FITC (clone MPO-7, Dako, Glostrup, Denmark), cyCD79a-PE (clone HM57, Dako, Glostrup, Denmark), CD34-PerCP-Cy5.5 (clone 8G12, BD Biosciences, San Jose, CA, USA), CD19-PE-Cy7 (clone J4.119, Beckman Coulter, Brea, CA, USA), CD3-APC-H7 (clone SK7, BD Biosciences, San Jose, CA, USA), cyCD3-Pacific Blue (clone UCHT1, BD Pharmingen, San Diego, CA, USA), CD7-APC (clone 124-1D1, Exbio Praha a.s., Vestec, Czech Republic), and CD45-Pacific Orange (clone HI30, Invitrogen, Carlsbad, CA, USA). Samples were processed and analyzed according to standardized procedures validated by the EuroFlow Consortium [11,17,20]. Flow cytometric acquisition was performed using FACSCanto II and FACSCanto 10-color flow cytometers (Becton Dickinson, San Jose, CA, USA) equipped with 405 nm, 488 nm and 633 nm lasers. Daily quality control and instrument standardization were performed using Cytometer Setup and Tracking (CS&T) beads according to EuroFlow SOPs. Additional monitoring of fluorescence signal stability was performed using Rainbow Calibration Particles.

A minimum of 50,000 events was acquired for each sample. Data acquisition was performed using FACSDiva 6.1 software (BD Biosciences), while data analysis was performed using Infinicyt 1.8-2.0 software (Cytognos/BD Biosciences). Detailed information regarding the antibodies included in the ALOT panel is provided in Supplementary Table S1. Representative examples of gating strategies and diagnostic orientation are shown in Supplementary Figures S1–S3.

### 2.3. Rationale for Marker Selection in ALOT Panel

The ALOT panel was designed to enable rapid discrimination between the major acute leukemia lineages by combining markers specific for myeloid, B-lymphoid, and T-lymphoid differentiation, as well as markers of cellular immaturity. Myeloperoxidase (MPO) is a key marker of myeloid lineage and allows for the identification of acute myeloid leukemia. CD19 and cytoplasmic CD79a (cyCD79a) are specific for B-cell lineage and are essential for the diagnosis of B-cell precursor ALL. CD3 and cytoplasmic CD3 (cyCD3) are highly specific markers of T-cell lineage, enabling identification of T-ALL. CD7, although not entirely lineage-specific, is frequently expressed in T-cell leukemias and some myeloid leukemias, supporting lineage assignment. CD34 is a marker of hematopoietic progenitor cells and reflects cellular immaturity, which is characteristic of acute leukemias. CD45, a pan-leukocyte marker, is crucial for gating strategies and allows for the distinction between leukemic blasts and normal hematopoietic cells based on CD45 expression and side scatter properties [17,21].

### 2.4. Results Analysis

Based on leukemia-specific immunophenotyping, each patient was assigned to one of the following diagnostic categories:

1. B-cell precursor acute lymphoblastic leukemia (BCP-ALL).
2. T-cell acute lymphoblastic leukemia (T-ALL).
3. Acute myeloid leukemia (AML).
4. Burkitt leukemia/lymphoma (B-AL).
5. Chronic myeloid leukemia (CML).
6. Myelodysplastic syndrome with excess blasts (MDS-EB).
7. Transient myeloproliferative syndrome (TMS).
8. Normal bone marrow findings.

Subsequently, all diagnoses were confirmed using multiparameter flow cytometry with extended disease-specific EuroFlow immunophenotypic panels, cytomorphology, cytogenetic studies, molecular testing and clinical findings when applicable. To evaluate the diagnostic performance of the ALOT screening test in acute leukemia, the following parameters were calculated: sensitivity, specificity, precision, accuracy, and negative predictive value. Sensitivity reflects the ability of the test to correctly identify patients with the disease (true positives), whereas specificity reflects the ability to correctly identify individuals without the disease (true negatives). Precision (positive predictive value) represents the proportion of true positive results among all positive results. Accuracy indicates the proportion of correctly classified cases among all evaluated cases. Negative predictive value represents the probability that patients with a negative test result truly do not have leukemia. The following formulas were applied:

1. Sensitivity = TP/(TP + FN).
2. Specificity = TN/(TN + FP).
3. Precision = TP/(TP + FP).
4. Accuracy = (TP + TN)/(TP + TN + FP + FN).
5. Negative predictive value (NPV) = TN/(TN + FN).

where

- TP (true positive)—the number of correctly identified leukemia cases.
- FN (false negative)—the number of cases not identified as leukemia by ALOT but confirmed by further testing.
- TN (true negative)—the number of correctly identified non-leukemia cases.
- FP (false positive)—the number of cases incorrectly identified as leukemia.

Because ALOT is designed as a screening and orientation tool rather than a fully comprehensive diagnostic assay, its performance was evaluated at two levels: (1) discrimination between abnormal and normal samples, and (2) exact diagnostic classification using a single-tube approach. Cases requiring additional immunophenotypic characterization were classified as indeterminate rather than false negative results.

## 3. Results

### 3.1. Study Cohort

Among the 254 patients analyzed using the ALOT screening panel, a definitive diagnosis was established in 244 cases. In the remaining 10 cases, additional immunophenotypic panels were required; however, none of these patients were ultimately classified as healthy. Normal bone marrow findings were identified in 20 cases. Within the study cohort, 186 cases of acute lymphoblastic leukemia (ALL) were identified, including 162 cases of B-cell precursor ALL (BCP-ALL) and 24 cases of T-cell ALL (T-ALL). In addition, 33 cases of acute myeloid leukemia (AML) and 5 cases of myelodysplastic syndrome with excess blasts (MDS-EB) were diagnosed. Less frequent diagnoses included Burkitt leukemia (BL, *n* = 5), chronic myeloid leukemia (CML, *n* = 2), and transient myeloproliferative syndrome (TMS, *n* = 3).

### 3.2. Diagnostic Performance of the ALOT Panel

The ALOT panel demonstrated excellent diagnostic performance as a screening and orientation tool. All cases of BCP-ALL, T-ALL, AML, and MDS were correctly identified at the initial stage, resulting in 100% sensitivity and specificity for these entities within the scope of diagnostic orientation (Table 2). In contrast, the ALOT panel did not provide sufficient immunophenotypic resolution for definitive classification of hematologic disorders such as Burkitt leukemia, chronic myeloid leukemia, and transient myeloproliferative syndrome, for which additional immunophenotypic staining was required in all cases. Importantly, these cases were not misclassified as normal, but rather recognized as abnormal and directed for further diagnostic work-up. In all abnormal cases, disease-specific extended immunophenotypic panels were subsequently applied according to EuroFlow recommendations in order to establish the final diagnosis and define leukemia-associated immunophenotypic characteristics.

Overall, the ALOT panel enabled correct initial diagnostic orientation in the majority of cases and significantly reduced the need for extended diagnostic panels in common leukemia subtypes.

### 3.3. Global Diagnostic Parameters

When evaluated as a screening tool for discrimination between abnormal and normal samples, the ALOT panel achieved 100% sensitivity and 100% specificity, as no pathological sample was classified as normal and no normal sample was classified as pathological. Positive predictive value, negative predictive value, and overall accuracy were also 100%. In terms of exact diagnostic classification using a single-tube approach, ALOT correctly classified 244 of 254 cases (96.1%). The remaining 10 cases (3.9%) required extension of the immunophenotypic panel for final diagnosis. Importantly, none of these cases were misclassified as normal, and therefore, they were considered indeterminate rather than false negative results.

## 4. Discussion

**Figure 1:**
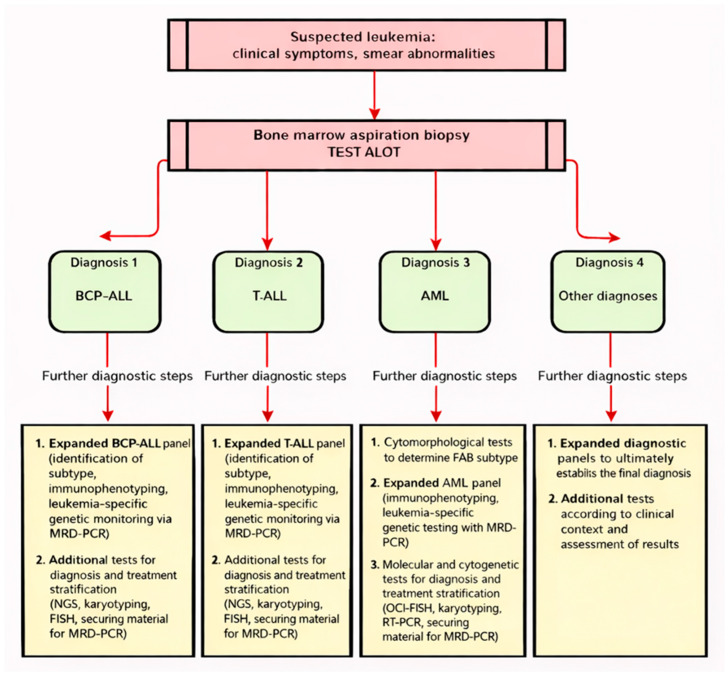
Schematic diagnostic algorithm for suspected leukemia [16,17,18,19].

Multiparameter flow cytometry is a key diagnostic tool in the evaluation of acute leukemias in both pediatric and adult patients [4,7]. It enables rapid and precise immunophenotypic characterization of leukemic cells, which is essential for accurate classification and prognostic stratification [18]. In addition to its diagnostic role, flow cytometry allows for the monitoring of treatment response, including minimal residual disease (MRD), which is critical for long-term disease control [6,19,22]. Owing to its ability to simultaneously assess multiple cellular markers, it is particularly useful in distinguishing between different hematologic malignancies at an early stage of the diagnostic process [4]. An ideal screening test should enable early detection of disease, thereby facilitating prompt initiation of appropriate therapy. The World Health Organization screening criteria emphasize that an effective screening test should combine high diagnostic performance with simplicity, reproducibility, accessibility, and cost-effectiveness [23]. These characteristics are particularly important in acute leukemia, where rapid diagnostic orientation may significantly accelerate the initiation of appropriate treatment. Such a test should be characterized by high sensitivity and specificity, as well as high positive and negative predictive values. In addition, it should be widely available, cost-effective, easy to perform, and reproducible across different laboratories. These criteria provide a useful framework for evaluating the clinical utility of screening tools in hematologic diagnostics. The rapid turnaround time and broad availability of flow cytometry support its use as an initial screening modality in suspected acute leukemia. The single-tube ALOT panel, developed by the EuroFlow Consortium and comprising a limited set of lineage-specific markers, appears to meet several criteria of an ideal screening test. The findings of the present study support its high diagnostic performance in the initial evaluation of pediatric acute leukemia [18,19]. A particular strength of the present study is the inclusion of a large consecutive pediatric cohort reflecting routine clinical practice, including both common acute leukemias and rare hematologic malignancies, as well as patients with normal bone marrow findings. In particular, ALOT proved highly effective in the initial orientation of common leukemia subtypes, such as ALL and AML, enabling rapid diagnostic orientation and facilitating the selection of appropriate extended immunophenotypic panels. By narrowing the differential diagnosis at the initial stage, the ALOT approach reduces the need for multiple parallel antibody combinations and supports a more efficient use of laboratory resources. Previous EuroFlow-based studies reported high diagnostic accuracy of standardized screening panels, typically exceeding 90% for major leukemia subtypes [18,19]. In this context, the performance observed in our cohort is consistent with these findings. According to van Dongen et al., the ALOT panel, as part of the EuroFlow standardization approach, plays a central role in rapid orientation of leukemia diagnostics and narrowing the differential diagnosis to specific subtypes [17]. In the present cohort, the ALOT panel achieved 100% sensitivity within the predefined diagnostic categories for BCP-ALL, T-ALL, AML, and MDS. These findings are in line with EuroFlow standardization studies, which also reported high sensitivity for these disease categories [19]. The absence of false-positive results in non-leukemic cases further underscores the high specificity of the method and supports its role as a reliable screening tool, consistent with data reported in the literature [18,19,24]. The ALOT panel includes markers selected to detect the most common leukemia subtypes; therefore, its diagnostic performance is inherently limited in rare entities. In our study, cases of Burkitt leukemia, CML, and transient myeloproliferative syndrome required additional immunophenotypic characterization beyond the initial ALOT panel. This observation is consistent with the biological characteristics of these disorders, which frequently require integration of additional immunophenotypic, cytogenetic, molecular, and clinical data for definitive diagnosis [24,25,26,27]. For example, Burkitt lymphoma/leukemia often requires more extensive immunophenotypic characterization than can be provided by a screening panel alone [24], whereas chronic myeloid leukemia is defined by the presence of the BCR::ABL1 fusion gene [26]. Similarly, transient myeloproliferative syndrome requires integration of clinical findings with cytogenetic and molecular features, particularly in patients with Down syndrome [27]. Therefore, screening panels should be viewed as an initial step in a comprehensive diagnostic algorithm rather than a substitute for disease-specific immunophenotypic, cytogenetic, and molecular investigations. Importantly, however, the preliminary information obtained from ALOT enabled targeted selection of extended diagnostic panels, thereby streamlining the diagnostic workflow and reducing unnecessary testing. An additional diagnostic challenge in flow cytometric evaluation of acute leukemias is represented by mixed phenotype acute leukemia (MPAL), which was not observed in the present cohort. MPAL is characterized by the co-expression of markers of more than one lineage, often requiring strict application of WHO classification criteria and extended immunophenotypic panels for accurate diagnosis [13,19,28]. In such cases, limited screening panels, including ALOT, may not provide sufficient resolution for definitive classification. Nevertheless, the use of an initial orientation tube may still facilitate early recognition of aberrant immunophenotypes and guide the selection of appropriate extended diagnostic panels [15,17,29]. A major strength of the ALOT approach lies in its standardization. Unlike locally developed flow cytometry panels, ALOT is based on protocols established and validated by the EuroFlow Consortium through extensive multicenter collaboration involving numerous diagnostic laboratories across Europe [30,31,32]. This approach has enabled the development of harmonized procedures for sample preparation, instrument setup, data acquisition, and interpretation, resulting in improved reproducibility and comparability of flow cytometric results between centers [30,31]. The panel is based on EuroFlow-validated protocols for sample preparation and data acquisition, which have been implemented in many leading centers worldwide. This ensures a high level of reproducibility and comparability of results across laboratories [18,19,31]. Furthermore, advanced analytical tools developed within the EuroFlow framework, such as principal component analysis (PCA) and database-guided automated gating (e.g., Compass), enhance data interpretation, reduce operator-dependent variability, and support rapid and objective classification of acute leukemias [11,18,19,20]. Taken together, the results of this study support the role of the ALOT panel as a highly effective screening tool for the rapid identification of common pediatric acute leukemias. Its implementation in routine clinical practice facilitates early initiation of therapy and improves the efficiency and cost-effectiveness of diagnostic workflows. In cases of rare or atypical hematologic malignancies, such as Burkitt leukemia or CML, ALOT should be complemented by extended flow cytometry panels and molecular analyses to achieve full diagnostic characterization and prognostic assessment [16,18,19]. Importantly, the interpretation of diagnostic performance metrics for ALOT requires consideration of its intended role as a screening and orientation tool. In this context, cases requiring additional immunophenotypic characterization should not be considered false negative results, as the panel correctly identified them as abnormal and directed further diagnostic work-up. Therefore, classical binary performance metrics should be interpreted separately from the rate of exact one-tube classification. The use of ALOT as a first-line screening tool supports a stepwise, algorithm-based diagnostic approach. A proposed diagnostic algorithm incorporating ALOT as the initial step is presented in Figure 1.

## 5. Conclusions

The results of this study support the clinical utility of the ALOT panel as a screening tool in the diagnosis of pediatric acute leukemia. The ALOT test proved particularly useful in the identification of ALL and AML, enabling rapid and accurate diagnostic orientation in the majority of cases. In this cohort, no false positive or false negative results among non-leukemic patients were observed, highlighting the high specificity and excellent negative predictive value of the ALOT panel, confirming its reliability as a rule-out tool in patients with suspected acute leukemia. When used as an initial diagnostic step, ALOT facilitates the targeted selection of subsequent disease-specific diagnostic panels and may help limit the extent of downstream immunophenotypic investigations. This approach may support a more efficient diagnostic workflow, although its impact on diagnostic time and cost-effectiveness was not formally evaluated in the present study. Although the ALOT panel was not sufficient for the definitive classification of rare entities such as CML, Burkitt leukemia, or transient myeloproliferative syndrome, it still provided valuable preliminary information that guided the selection of additional diagnostic tests and marker panels required to establish the final diagnosis. Importantly, an effective screening test should be widely available, cost-effective, relatively simple to perform, and characterized by high predictive value and diagnostic reliability. The findings of this study indicate that the ALOT panel fulfills these key criteria, supporting its role as a robust and clinically valuable screening tool in routine pediatric hematology practice.

## Figures and Tables

**Table 1:** Characteristics of study population.

Leukemia Type (WHO Classification)	Number of Cases	Females	Males	Age Range (Years)
BCP-ALL	162	82	80	0–18
T-ALL	24	6	18	1–18
AML	33	13	20	0–18
MDS	5	1	4	7–10
BL	5	1	4	8–13
CML	2	1	1	16–18
TMS	3	0	3	0–1/12
Normal bone marrow	20	6	14	0–18

**Table 2:** Performance of the ALOT panel in the orientation of hematologic disorders.

Diagnosis	*n*	Correct Diagnostic Orientation	Additional Testing	Orientation Rate (%)
BCP-ALL	162	162	0	100%
T-ALL	24	24	0	100%
AML	33	33	0	100%
MDS	5	5	0	100%
BL	5	0	5	0%
CML	2	0	2	0%
TMS	3	0	3	0%
Normal bone marrow	20	20	0	100%
All cases	254	244	10	96.1%

## Data Availability

The data presented in this study are available from the corresponding author upon reasonable request. The data are not publicly available due to privacy and ethical restrictions.

## Supplementary Materials

The following supporting information can be downloaded at: https://www.mdpi.com/article/10.3390/cancers18132023/s1. Figure S1: Representative gating strategy used for the identification of B-cell precursor acute lymphoblastic leukemia (BCP-ALL). Figure S2: Representative gating strategy used for the identification of T-cell acute lymphoblastic leukemia (T-ALL). Figure S3: Representative gating strategy used for the identification of acute myeloid leukemia (AML). Table S1: Antibodies included in the EuroFlow Acute Leukemia Orientation Tube (ALOT).

## Abbreviations

The following abbreviations are used in this manuscript:

ALOT	Acute Leukemia Orientation Tube
ALL	Acute Lymphoblastic Leukemia
BCP-ALL	B-cell Precursor Acute Lymphoblastic Leukemia
T-ALL	T-cell Acute Lymphoblastic Leukemia
AML	Acute Myeloid Leukemia
MDS	Myelodysplastic Syndrome
MDS-EB	Myelodysplastic Syndrome with Excess Blasts
BL	Burkitt Leukemia/Burkitt Lymphoma
B-AL	Burkitt Acute Leukemia
CML	Chronic Myeloid Leukemia
TMS	Transient Myeloproliferative Syndrome
MPAL	Mixed Phenotype Acute Leukemia
MRD	Minimal Residual Disease
PCA	Principal Component Analysis
TP	True Positive
TN	True Negative
FP	False Positive
FN	False Negative
NPV	Negative Predictive Value
PPV	Positive Predictive Value
CD	Cluster of Differentiation
WHO	World Health Organization
