# Supplementary material for: Diagnostic Performance of the EuroFlow Acute Leukemia Orientation Tube (ALOT) in Pediatric Acute Leukemia: A Single-Center Experience
Source: Cancers (Basel). 2026 Jun 23;18(13):2023. doi: 10.3390/cancers18132023 (PMC13359837; doi:10.3390/cancers18132023)

Supplementary Figure S2. Representative gating strategy and diagnostic orientation of B-cell precursor acute lymphoblastic leukemia (BCP-ALL) using the EuroFlow ALOT panel.

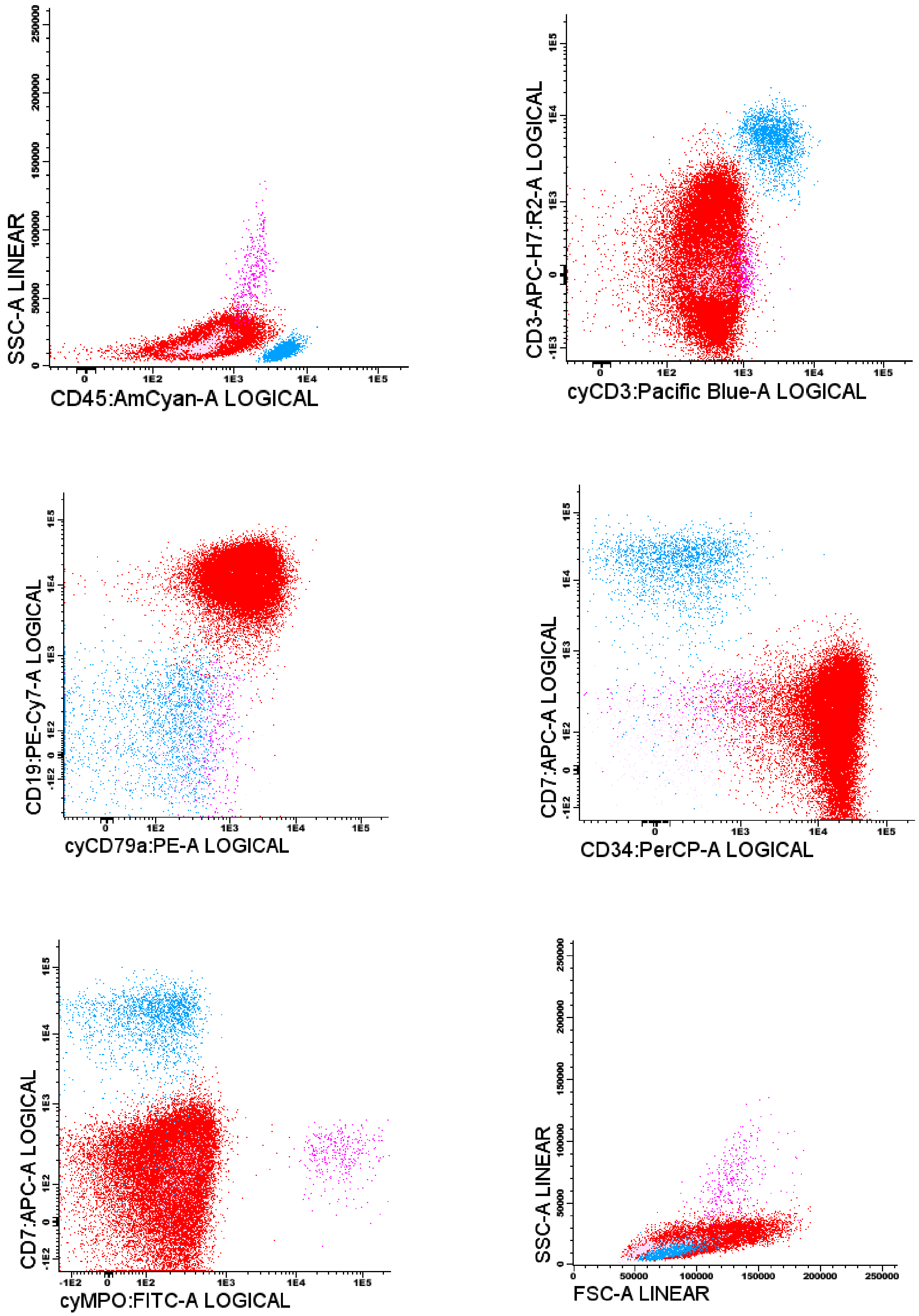

Supplement: Supplementary file 1 [file cancers-18-02023-s001.zip › suplementary Figure S2.pdf]
