# Supplementary material for: Diagnostic Performance of the EuroFlow Acute Leukemia Orientation Tube (ALOT) in Pediatric Acute Leukemia: A Single-Center Experience
Source: Cancers (Basel). 2026 Jun 23;18(13):2023. doi: 10.3390/cancers18132023 (PMC13359837; doi:10.3390/cancers18132023)
Supplement: Supplementary file 1 [file cancers-18-02023-s001.zip › suplementary Table S1.pdf]

Supplementary Table S1. Antibodies included in the EuroFlow Acute Leukemia Orientation Tube (ALOT).

| Marker | Fluorochrome | Clone | Manufacturer |
|---|---|---|---|
| cyMPO | FITC | MPO-7 | Dako |
| cyCD79a | PE | HM57 | Dako |
| CD34 | PerCP-Cy5.5 | 8G12 | BD Biosciences |
| CD19 | PE-Cy7 | J4.119 | Beckman Coulter |
| CD7 | APC | 124-1D1 | Exbio |
| CD3 | APC-H7 | SK7 | BD Biosciences |
| cyCD3 | Pacific Blue | UCHT1 | BD Pharmingen |
| CD45 | Pacific Orange | HI30 | Invitrogen |
